# Cross-species single-cell landscapes identify the pathogenic gene characteristics of inherited retinal diseases

**DOI:** 10.3389/fgene.2024.1409016

**Published:** 2024-07-11

**Authors:** Hualei Hu, Fei Liu, Pan Gao, Yuwen Huang, Danna Jia, Jamas Reilly, Xiang Chen, Yunqiao Han, Kui Sun, Jiong Luo, Pei Li, Zuxiao Zhang, Qing Wang, Qunwei Lu, Daji Luo, Xinhua Shu, Zhaohui Tang, Mugen Liu, Xiang Ren

**Affiliations:** ^1^ Key Laboratory of Molecular Biophysics of Ministry of Education, College of Life Science and Technology, Huazhong University of Science and Technology, Wuhan, China; ^2^ State Key Laboratory of Freshwater Ecology and Biotechnology, Institute of Hydrobiology, The Innovative Academy of Seed Design, Hubei Hongshan Laboratory, Chinese Academy of Sciences, Wuhan, China; ^3^ University of Chinese Academy of Sciences, Beijing, China; ^4^ Department of Biological and Biomedical Sciences, Glasgow Caledonian University, Glasgow, Scotland

**Keywords:** inherited retinal disease, single-cell RNA sequencing, retina, cross-species, transcription factor regulatory network

## Abstract

**Introduction:**

Inherited retinal diseases (IRDs) affect ∼4.5 million people worldwide. Elusive pathogenic variants in over 280 genes are associated with one or more clinical forms of IRDs. It is necessary to understand the complex interaction among retinal cell types and pathogenic genes by constructing a regulatory network. In this study, we attempt to establish a panoramic expression view of the cooperative work in retinal cells to understand the clinical manifestations and pathogenic bases underlying IRDs.

**Methods:**

Single-cell RNA sequencing (scRNA-seq) data on the retinas from 35 retina samples of 3 species (human, mouse, and zebrafish) including 259,087 cells were adopted to perform a comparative analysis across species. Bioinformatic tools were used to conduct weighted gene co-expression network analysis (WGCNA), single-cell regulatory network analysis, cell–cell communication analysis, and trajectory inference analysis.

**Results:**

The cross-species comparison revealed shared or species-specific gene expression patterns at single-cell resolution, such as the stathmin family genes, which were highly expressed specifically in zebrafish Müller glias (MGs). Thirteen gene modules were identified, of which nine were associated with retinal cell types, and Gene Ontology (GO) enrichment of module genes was consistent with cell-specific highly expressed genes. Many IRD genes were identified as hub genes and cell-specific regulons. Most IRDs, especially the retinitis pigmentosa (RP) genes, were enriched in rod-specific regulons. Integrated expression and transcription regulatory network genes, such as congenital stationary night blindness (CSNB) genes *GRK1*, *PDE6B*, and *TRPM1*, showed cell-specific expression and transcription characteristics in either rods or bipolar cells (BCs). IRD genes showed evolutionary conservation (*GNAT2*, *PDE6G*, and *SAG*) and divergence (*GNAT2*, *MT-ND4*, and *PDE6A*) along the trajectory of photoreceptors (PRs) among species. In particular, the Leber congenital amaurosis (LCA) gene *OTX2* showed high expression at the beginning of the trajectory of both PRs and BCs.

**Conclusion:**

We identified molecular pathways and cell types closely connected with IRDs, bridging the gap between gene expression, genetics, and pathogenesis. The IRD genes enriched in cell-specific modules and regulons suggest that these diseases share common etiological bases. Overall, mining of interspecies transcriptome data reveals conserved transcriptomic features of retinas across species and promising applications in both normal retina anatomy and retina pathology.

## 1 Introduction

Inherited retinal diseases (IRDs) are a complex group of heterogeneous and mainly monogenic phenotypes (Schneider et al., 2022). Patients with IRDs exhibit a wide range of clinical manifestations, varying from legal blindness in severe forms of retinal degeneration, such as Leber congenital amaurosis (LCA), to less severe retinal dysfunctions, such as congenital stationary night blindness (CSNB) (Hohman, 2017). Retinitis pigmentosa (RP) is the most common inherited retinal degeneration disease. Other common IRDs include cone–rod dystrophies (CRDs), macular degeneration (MD), and CSNB. A number of genes have been involved in the etiology of IRDs, indicating their high genetic heterogeneity. Traditional approaches to mutation identification are limited, resulting in low diagnosis rates.

**FIGURE 1:**
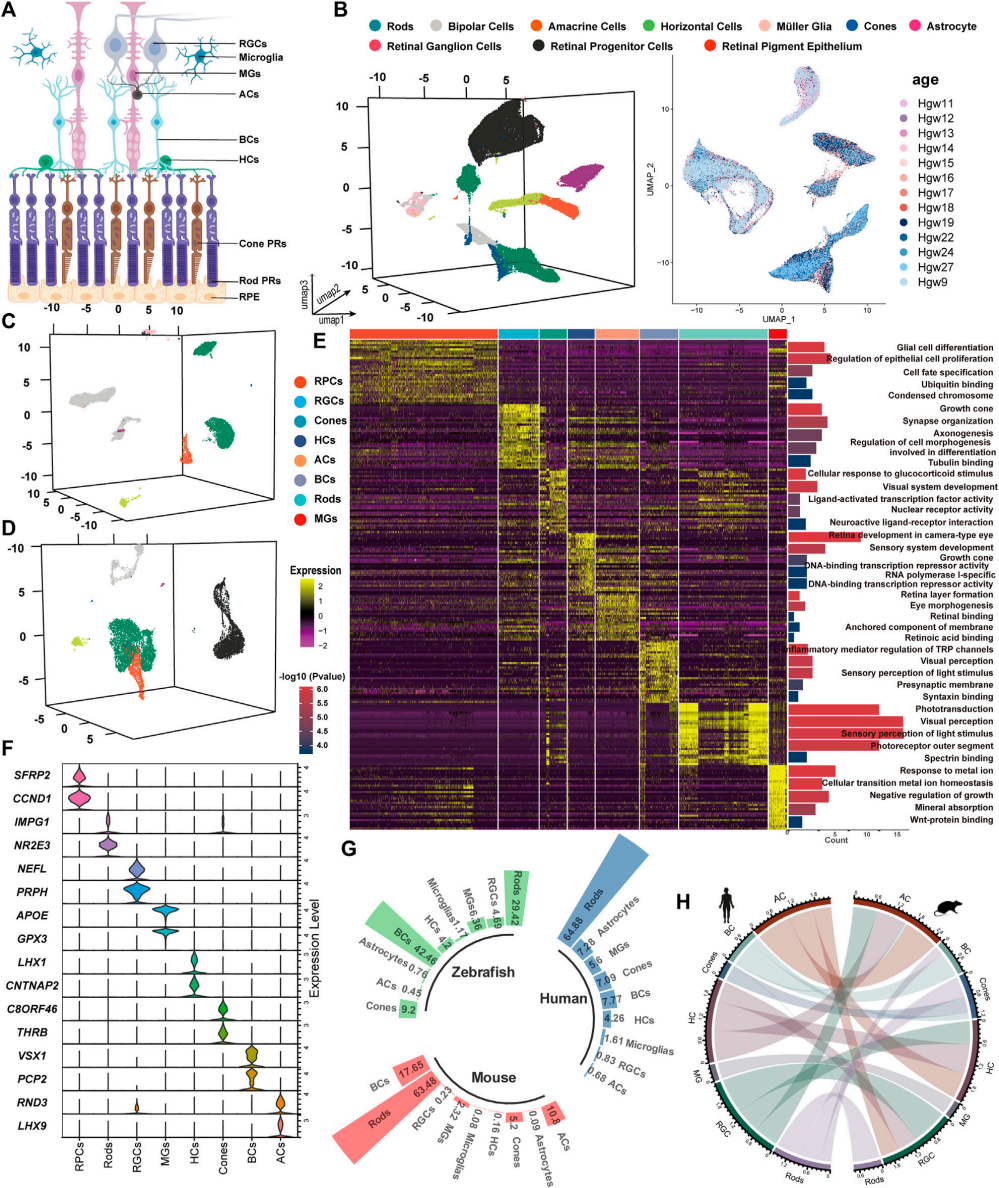
Unsupervised clustering for single-cell transcriptomic analysis to identify retinal cell classes. **(A)** Sketch of retinal sections showing the main cell types. **(B–D)** tSNE cluster results of the expression profile of 86,962 developing human whole-retinal cells **(B)**, 9,070 developing human macula cells **(C)**, and 11,005 developing human periphery cells **(D)**. **(E)** Heatmap of DEGs from each cell type and the GO term enrichment of each set of DEGs. For visualization, the top five GO terms with the lowest *p*-value were used. Each column and row represent a single cell and gene, respectively. **(F)** Average expression of known marker genes in each cell class. **(G)** Statistics on the proportion of major cell types in the adult human whole retina, adult mouse whole retina, and adult zebrafish whole retina. **(H)** Transcriptional pattern correlation of major retinal types in humans and mice. The top 3,000 highly variable genes were extracted for correlation analysis, with the correlation coefficient >0.6 reserved for presentation, and the line width indicated correlation.

The development of the retina involves the differentiation and migration of retinal progenitor cells (RPCs) into various cell types, including rod photoreceptor cells (rods), cone photoreceptor cells (cones), horizontal cells (HCs), amacrine cells (ACs), bipolar cells (BCs), Müller glial (MG) cells, and retina ganglion cells (RGCs) (Figure 1A) (Chow and Lang, 2001; Marquardt and Gruss, 2002; Marquardt, 2003; Henry Klassen and Young, 2004; Agathocleous and Harris, 2009). Humans possess a fovea centralis, a structure dominated by morphologically distinct cones and Müller cells (Yuodelis and Hendrickson, 1986; Bringmann et al., 2018). The development of the retina necessarily relies on intercellular communication for the coordinated differentiation and localization of cell types. Ligand–receptor interactions and co-expressed gene modules can be valuable clues to understand the physiology of vision and the etiology of IRDs.

Given the diverse variety of clinical symptoms and the involvement of dozens of cell types and hundreds of genes, it is critical to obtain a comprehensive understanding of the cell–gene regulatory network (GRN) for pathology and clinical diagnosis of IRDs. Studies using single-cell RNA sequencing (scRNA-seq) have identified alterations in gene expression in retinas (Voigt et al., 2019a; Voigt et al., 2019b; Hu et al., 2019; Liang et al., 2019; Menon et al., 2019; Yan et al., 2020). However, there is no comprehensive view of IRD gene heterogeneity and the GRN of multiple species at single-cell resolution. The regulons among species help explain the conservatism and difference of cell destiny across species (Datta et al., 2018; Feldker et al., 2020). Cross-species characterization of IRD genes at the single-cell level will aid in exploring and comprehending the similarities and differences among multiple species in the occurrence, development, and treatment of IRDs.

In this study, we incorporated scRNA-seq data of humans, mice, and zebrafish to gain an in-depth understanding of IRDs and biological processes in the view of evolutionary developmental biology. We constructed a weighted co-expression gene network, a single-cell transcription factor (TF) regulatory network, an intercellular ligand–receptor communication network, and pseudotime analysis that delineated IRDs engaged in photoreceptor differentiation state. Furthermore, cross-species disparities and commonalities in retinal cells have been identified. In short, we characterized IRD genes from different perspectives, which can serve to elucidate the etiology of these IRDs on a molecular basis and provide a theoretical basis for the development of therapeutic interventions.

## 2 Materials and methods

### 2.1 Datasets and sample characteristics

The retina scRNA-seq datasets of the three model organisms, namely, *Homo sapiens* (human), *Mus musculus* (mouse), and *Danio rerio* (zebrafish), were downloaded from Gene Expression Omnibus (GEO) and ArrayExpress. These datasets include developing human retina (GSE138002), adult human retina (E-MTAB-7316), developing mouse retina (GSE118614), adult mouse retina (GSE132229), and developing zebrafish and adult zebrafish retina (GSE122680 and GSE160140, respectively) (Clark et al., 2019; Heng et al., 2019; Lukowski et al., 2019; Lu et al., 2020; Xu et al., 2020; Liu et al., 2022). Specifically, adult human retinal samples, including the whole retina, macular region, and peripheral region, were separated for further analysis.

### 2.2 Ortholog gene selection

Homologous gene lists were downloaded from Ensembl BioMart. An aggregated table of “meta-genes” was created to account for gene paralogs and gene duplication events. Each meta-gene may include all gene symbols homologous to a specific human gene (Supplementary Table S1).

### 2.3 Identification of major cell types of the retinal single-cell transcriptome

The raw unique molecular identifier (UMI) count matrices were converted into a Seurat object using Seurat (v5.0.1) of the R package (Hao et al., 2024). Batch effects among the samples were alleviated using Harmony (Korsunsky et al., 2019). Cells with UMI numbers <1,000 or with feature counts <200 or with mitochondrial percentage >10 were considered low-quality cells and removed. The resulting datasets were normalized using the ScaleData function, and principal component analysis (PCA) was applied. The main cell clusters were identified using the FindClusters function, and 30 PCs were used in subsequent cell cluster analysis with a resolution of 0.8. They were then visualized using 3D t-distributed stochastic neighbor embedding (tSNE) plots. The cell barcode information of datasets was added to categorize every cell into a known biological cell type. Marker genes and differentially expressed genes (DEGs) were identified using the FindConservedMarkers and Findallmarker functions. |avg_log2FC| > 1 and Bonferroni-adjusted *p*-value (p_val_adj) < 0.05 were the screening threshold of significantly DEGs. We performed Gene Set Enrichment Analysis (GSEA) using clusterProfiler of the R package (Subramanian et al., 2005; Yu et al., 2012).

### 2.4 Construction of the co-expression module network to infer IRD gene expression characteristics

A total of 277 IRD genes selected from RetNet (https://sph.uth.edu/retnet/) through manual text mining (Supplementary Table S2) included the top 2,000 highly variable genes as input in our gene set. Weighted gene co-expression network analysis (WGCNA) was conducted by scWGCNA to identify functional modules in the co-expression network (Langfelder and Horvath, 2008; Morabito et al., 2021). An adjacency matrix was generated using a soft threshold (β = 4) to reach a scale-free topology. Average gene significance (GS) was calculated to identify the correlation between module eigengenes and a certain cell type. For each gene, Pearson’s correlation between the gene and module eigengene was calculated as module membership (MM). We screened for the hub genes of each module using the threshold |GS| > 0.2 and |MM| > 0.8. The interactive network was visualized using Cytoscape v3.9.0 (Shannon et al., 2003).

### 2.5 Construction of the regulatory network for inferring IRD gene-associated regulons

Single-Cell rEgulatory Network Inference and Clustering (SCENIC) enabled us to reconstruct a GRN based on co-expression and DNA motif analysis (Aibar et al., 2017). Simple regulons comprise TFs and the set of genes they regulate. SCENIC was performed on 2,000 cells randomly selected from each of the human and mouse whole retina to identify cell-specified regulons. In addition, hg19-500bp-upstream-10species and mm9-500bp-upstream-7species databases were served as RcisTarget (Herrmann et al., 2012; Imrichova et al., 2015). Regulon activity and regulon OFF/ON status were determined according to the area under the curve (AUC) score.

### 2.6 Identification of cell-specific signals using ligand–receptor communication analysis

Cell–cell communication analysis was performed using CellPhoneDB (Efremova et al., 2020). Ligand–receptor complexes play crucial roles in development, differentiation, inflammation, and other processes by orchestrating various biological actions. The CellPhoneDB results were visualized using the ggplot2 package.

### 2.7 Trajectory inference and trajectory alignment

Trajectory inference of photoreceptors (PRs) and BCs was performed using Monocle 2.24.0 and Monocle 3 1.3.1 (Trapnell et al., 2014; Qiu et al., 2017). After creating a Monocle object, we performed the analysis on the top 2,000 DEGs as ordering genes. The “DDTree” method was used for dimensionality reduction. Cluster and period annotations were projected on the inferred trajectories. Furthermore, Monocle developed BEAM to assess branch-dependent gene expression by formulating the problem as a contrast between the two negative binomial GLMs.

The list of data and method information was generated (Supplementary Methods).

## 3 Results

### 3.1 Single-cell expression atlas of the retina

By comparing the single-cell transcriptome, we analyzed a total of 259,087 cells and found that the correlation among most retinal cell types across species was significantly higher than that among cell types from different categories. Following quality control filtering, 107,037 cells from developing retinas were included, with 86,962 cells originating from the whole retina (Figure 1B), 9,070 cells (8.5%) from the macula, and 11,005 cells (10.3%) from the periphery (Figures 1C,D). Additionally, 83,994 cells from developing mouse retinas were categorized into 9 major retinal cell types, while 32,866 cells derived from 8 developing zebrafish retinas were classified into 10 cell types (Supplementary Figure S1A). Adult retinal samples from 3 species were divided into 8 major cell types, comprising 6,143 (human), 22,186 (mouse), and 4,861 (zebrafish) cells (Supplementary Figure S1B).

The top five GO terms enriched by each cell cluster were consistent with our current knowledge about the retina (Figure 1E). The known marker genes for retinal cell types showed a cluster-specific expression pattern (Figure 1F). Cross-species analysis revealed a highly conserved proportion of retinal cell types (Figure 1G). BCs and MGs demonstrated the highest degree of interspecific conservation between humans and mice, while there are obvious differences in the comparative analysis of GO terms of the top 30 markers among the three species (Figure 1H, Supplementary Figure S1C). A pairwise comparison between humans and other species showed that differences in the expression level of markers exist among species. In zebrafish MGs, *atoh7* is the most significantly highly expressed gene, while *GPX3* and *DKK3* are highly expressed genes in the human and mouse retina, respectively. The expression levels of IRDs (*SAG*, *RGR*, *ITM2B*, *etc*.) varied across species (Supplementary Figure S1A, Supplementary Figures S2A,B). These expression differences may contribute to phenotypic variations between different species. For instance, the mouse model with an *APOE* mutation recapitulated the clinical feature of AMD patients rather than the zebrafish model (Fletcher et al., 2014; Vessey et al., 2022). Our results provide a basis for constructing IRD models. Notably, the stathmin family genes, stmn1a/b (*STMN1*_homo1/2) and *stmn2a/b* (*STMN2*_homo1/2), were highly expressed in zebrafish MGs. *GPX3*, *APOE*, and *GADD45B* were highly expressed in human MGs, while *Dkk3* (*DKK3*_homo1/2), *Vim* (*VIM*_homo1/2), and *Junb* (*JUNB*_homo1/2) were highly expressed in mouse MGs.

### 3.2 Cell type-based expression of IRD genes

**FIGURE 2:**
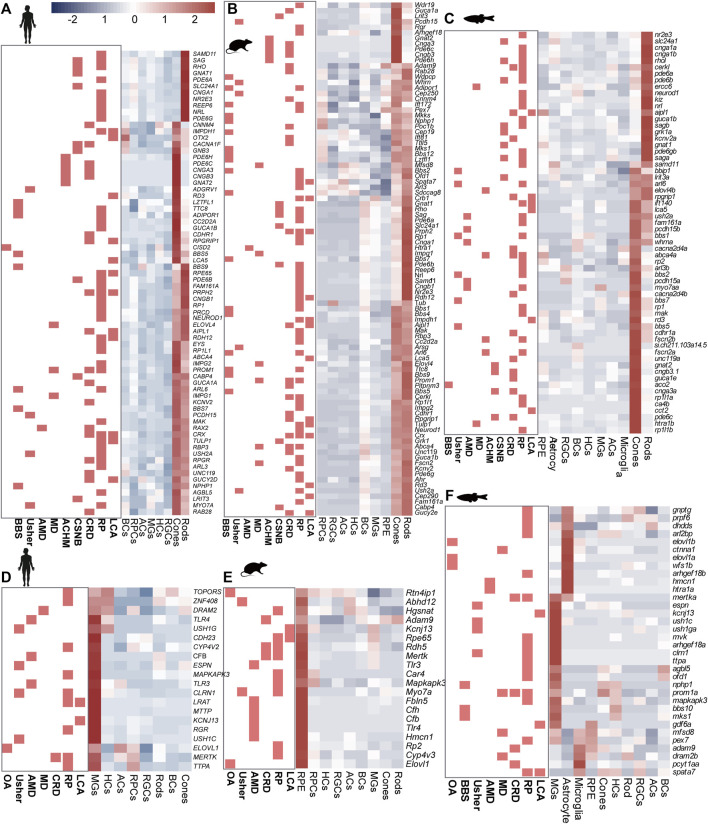
Expression heterogeneity of IRD gene in the retinal cells of three species. IRD genes highly expressed in **(A)** photoreceptors (human), **(B)** photoreceptors (mouse), **(C)** photoreceptors (zebrafish) **(D)** MGs (human), **(E)** RPCs (mouse), **(F)** MGs, astrocyte cells, and microglial cells (zebrafish).

Significant GO terms of IRD genes included cell development, differentiation, cell homeostasis, metabolic processes, and ion transport (Supplementary Figure S2C and Supplementary Table S3). Among these genes, most RP genes shared high homology across species and displayed similar expression patterns. For instance, the ceramide kinase-like (*CERKL*) gene showed high expression levels in the rods of humans, mice, and zebrafish. Additionally, we observed that certain genes exhibited species-specific expression, such as *Fam161a* and *NEUROD1* (Figures 2A–C), and differences in cell-specific expression patterns among species, such as *CFB* and *Elovl1* (Figures 2D–F, Supplementary Figures S2D,E).

Most CRD genes (such as *GNAT2* and *PDE6H*) are mainly expressed in cones and rods, and the expression level of cones is higher than that of rods, which is conservative in humans, mice, and zebrafish. However, there were also a few genes that showed species-specific expression. For instance, in humans and mice, the *AIPL1* gene was highly expressed in both cones and rods, while it was highly expressed in the RPCs and rods in zebrafish (Supplementary Figures S2D,E).

In addition to the rods and cones, MGs were also identified as significant sites for the distribution of IRD genes in humans and zebrafish, as opposed to mice. PR degeneration in geographic atrophy or choroidal neovascularization can be triggered during the onset of AMD (Deng et al., 2022). The complement pathway, which plays a crucial role in recognizing and mediating the removal of pathogens, debris, and dead cells, has been reported to be associated with AMD (Liu et al., 2010). Among the complement pathway genes, *C2*, *C3*, and *CFB* were highly expressed in MGs, highlighting the previously underestimated effect of MGs on retinal complement homeostasis (Figures 2D,F). However, we also identified that *CFH*, a complement pathway gene, was mainly expressed in BCs. Moreover, we observed that CSNB genes were highly expressed in either rods (e.g., *GRK1* and *PDE6B*) or BCs (e.g., *GPR179*, *NYX*, and *TRPM1*), whereas *CACNA1F* and *GNB3* were highly expressed in both BCs and PRs in humans (Figure 2A). These results showed that IRD gene expression exhibits cell specificity in humans, suggesting that different mutant genes leading to the same IRDs may be associated with their expression location in the retina. This insight provides clues for the subsequent exploration of the pathological mechanism of IRDs.

### 3.3 Identification of the heterogeneity of gene co-expression modules for IRDs using WGCNA

**FIGURE 3:**
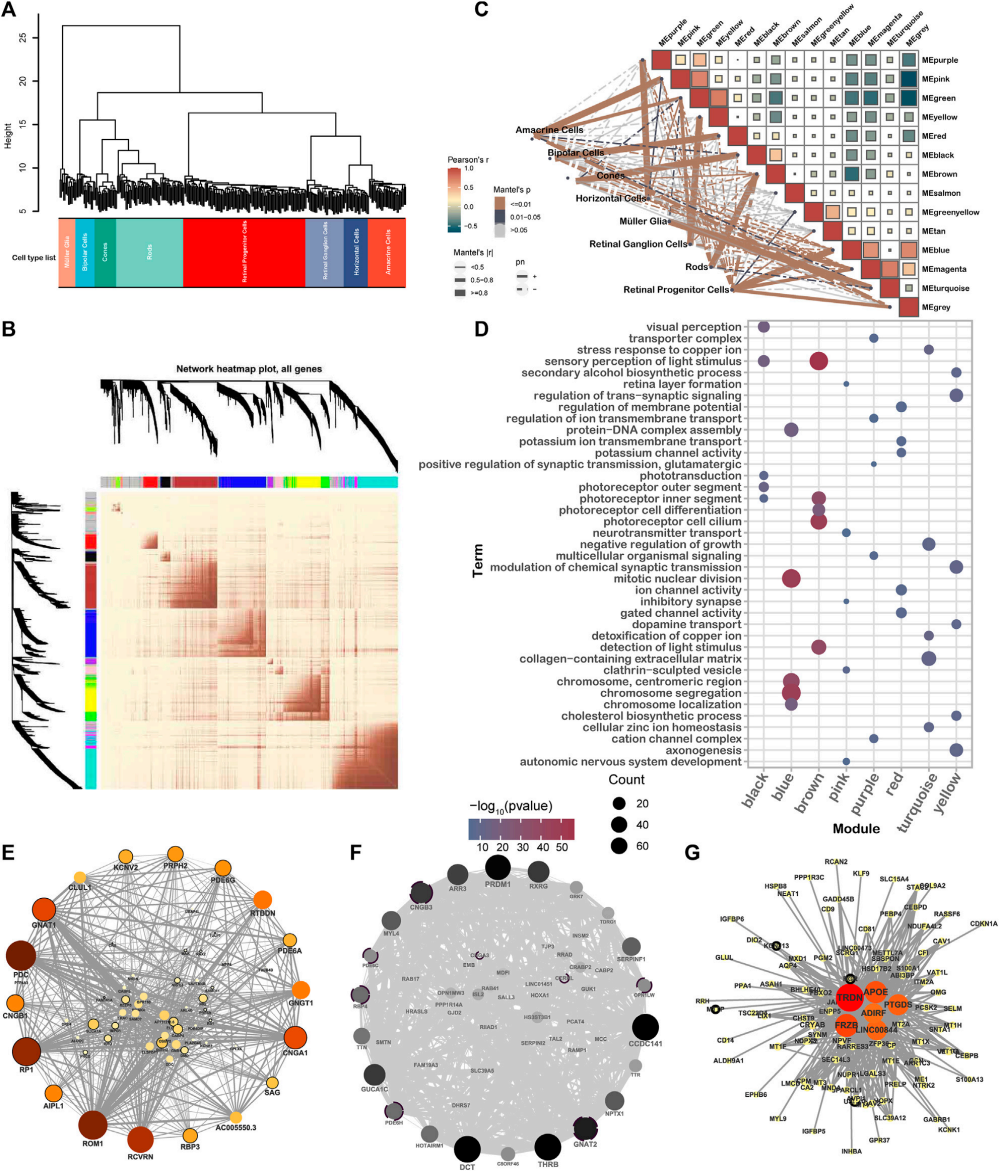
WGCNA reveals the gene network module and hub genes of different retinal cell types. **(A)** Clustering dendrogram of 283 samples. Samples were clustered according to the similarity of gene expression. **(B)** Heatmap visualization gene network based on the topological overlap matrix (TOM). Color depth represents the degree of overlap. **(C)** Module–module and module–cell type correlation statistical analysis, in which the color of the square represents the correlation of modules, the solid line and the dotted line represent positive and negative correlation, respectively, and the color and the thickness of the line represent the significance and correlation of module–cell type, respectively. **(D)** GO term enrichment by eigengenes of different modules. **(E–G)** Network and hub genes for brown **(E)**, black **(F)**, and yellow **(G)** modules. Hub genes were identified from the module genes using a degree analysis method. The depth of the color indicates the rank of the hub genes from low to high.

Cells were categorized into eight types based on their co-expression of the genes distinguished among these samples (Figure 3A). Subsequently, , 13 modules were identified according to WGCNA, with sizes ranging from 14 genes (salmon) to 348 genes (turquoise) (Figure 3B, Supplementary Figure 2F). Eight of these modules were highly correlated with retinal cell types: HCs (purple), ACs (pink), MGs (yellow), BCs (red), cones (black), rods (brown), RPCs (blue), and RGCs (turquoise) (Figure 3C, Supplementary Figure 3A).

The modules were identified to have significant associations with the recognized function of retinal cells (Figure 3D). Furthermore, many hub genes were identified among IRD genes, indicating a strong interconnection between different IRD genes. As shown n Figure 3E, *GNAT1* was recognized as a hub gene in the brown module. Similarly, *GNAT2*, *PDE6H*, *OPN1LW*, *RBP4*, *PDE6C*, and *CNGB3* were identified in the black module. It is worth noting that *GNAT2*, *PDE6H*, *PDE6C*, and *CNGB3* have been associated with ACHM, an IRD characterized by impaired cone PR function. Additionally, *MTTP*, *USH1C*, *KCNJ13*, and *RGR* were found in the yellow module (Figures 3E–G).

### 3.4 IRD genes were involved in the cell-specific regulatory network

**FIGURE 4:**
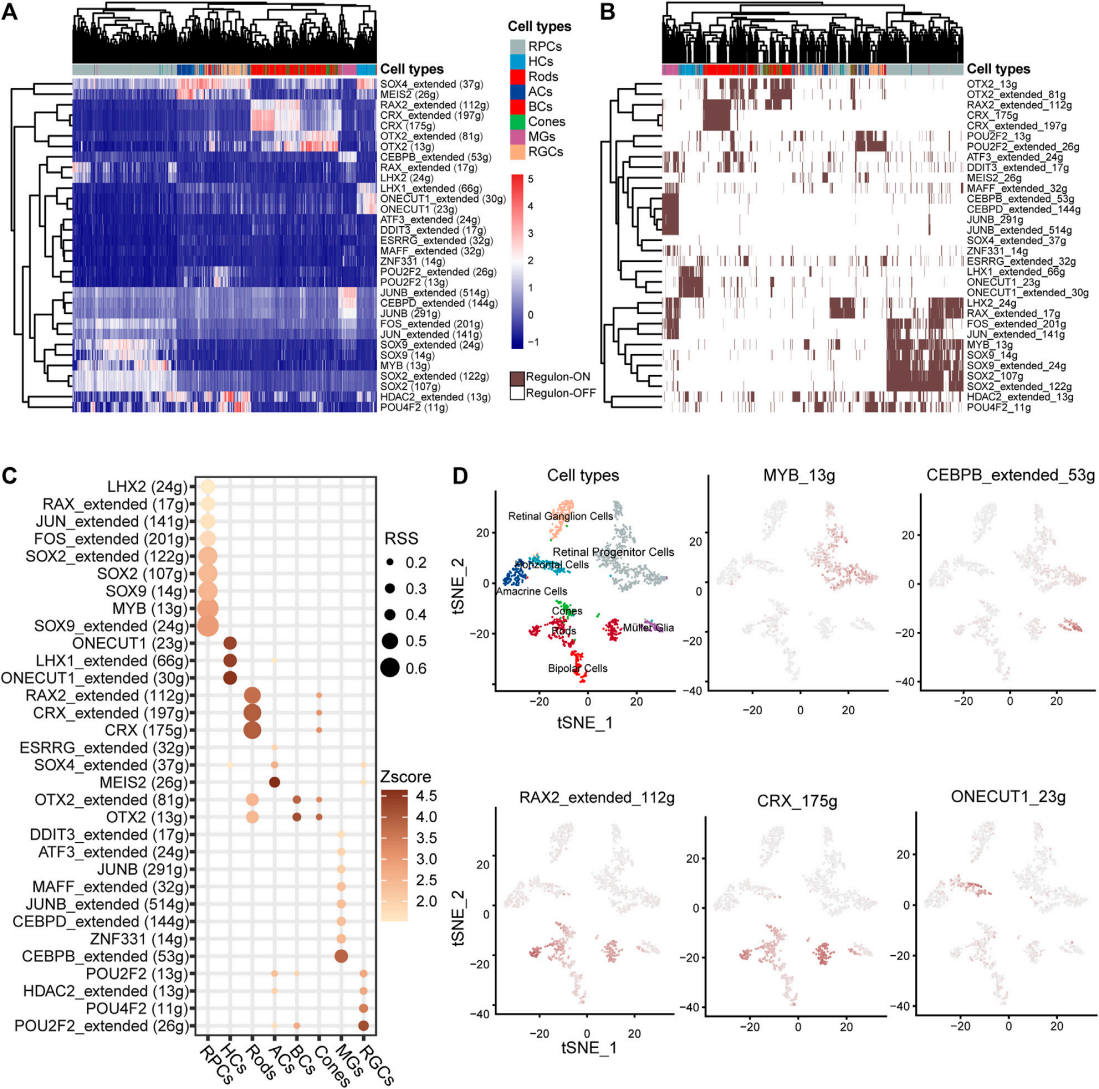
Identification of the TF regulation network of human retinal cells based on SCENIC. **(A)** Heatmap of the area under curve (AUC) score estimated by SCENIC. Colors distinguished the cell clusters of the retina. **(B)** Heatmap of the ON/OFF status of regulons. Brown/white indicates ON/OFF status. **(C)** Dot plot of the regulon specificity score (RSS) for the retinal core cell cluster. **(D)** tSNE plot of the AUC score for the major regulons of the retina.

TFs play a crucial role in determining cell fate and directly govern the transcription patterns. We discovered 32 cell-specific regulons (Figures 4A,B), many of which encompass IRD genes. Among the 44 IRD genes identified in the cell-specific regulons, 55% (22) were RP genes distributed in the rod-specific regulons (including *CRX* and *RAX2* regulons). The functions of RP genes in the *CRX* regulon include visual system development and PR outer-segment formation (Supplementary Figure 3C). Patients with CSNB are characterized by the dysfunction of rods and impaired signaling from PRs to BCs (Varin et al., 2021). Our findings indicate that CSNB genes are predominantly present in rod- or BC-specific regulons, such as *SLC24A1* in the *CRX* regulon, *GNB3* in the *RAX2* regulon, and *GRM6* in the *OTX2* regulon (Figure 4C, Supplementary Figure 2E), whereas *GRMP* was specifically highly expressed only in BCs (Figure 2A).

The *MYB* regulon was identified as the RPC-specific regulon with target genes *NR2E1*, *ZHX2*, *LAMA1*, and *EPHB2* associated in the eye development term. The *CEBPB* regulon was identified as MG-specific with target genes *CLU*, *TIMP3*, *VEGFA*, etc*.* (Figure 4C, Supplementary Figure 3C, Supplementary Table S4). We also found the specificity of TF regulons such as that *ONECUT1* and *LHX* specific in HCs, *RAX2* and CRX in rods, *MEIS2* in ACs, *ZNF331* and *CEBPB* in MGs, and *POU2F2* in RGCs (Figures 4C,D). Consistent with human results, the IRD gene *Crx* and *Otx2* regulons were identified as PR-specific in mice (Supplementary Figures 4A–C). The *Lhx9* regulon was identified as AC-specific, and the *Pou4f2* regulons were identified as HC- and BC-specific, while *Sox4* showed high AUC scores specific for both ACs and HCs (Supplementary Figures 4A, D–F). Remarkably, many TFs from the AP-1 family were identified as members of cell-specific regulons. The AP-1 complex is known to play an important role in neuron protection (Feldker et al., 2020). Regulons involving *JUNB*, *MAFF*, and *ATF3* were specific to MGs, while the *JUN* regulon was specific to RPCs. The AP-1 family-involved regulons (*Jun*, *Junb*, and *Fos*) were also identified in the mouse retina (Supplementary Figures 4G–I).

### 3.5 Complex intercellular communication networks among retinal cell types

**FIGURE 5:**
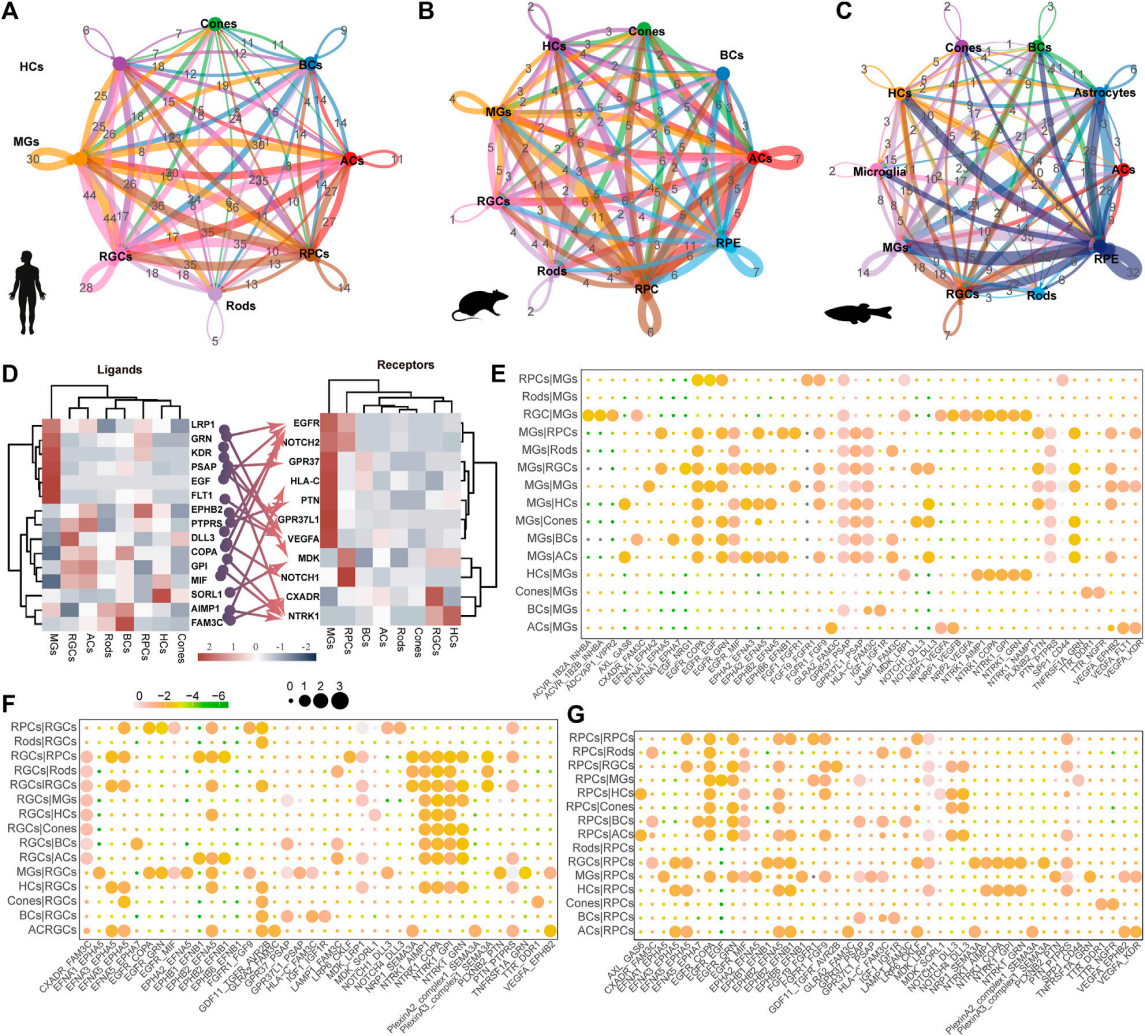
Ligand–receptor-based interactions between human retinal cells. **(A–C)** Intercellular communication ability among retinal cells of humans **(A)**, mice **(B)**, and zebrafish **(C)**. Line colors represent ligands expressed by retinal cells marked by the same color. Lines connect to the cells expressing the corresponding receptors. Line thickness is proportional to the number of ligands. Loops indicate autocrine circuits. **(D)** Heatmap shows the gene expression levels of receptor–ligand pairs involved in interactions between different clusters in the human retina. **(E–G)** Overview of selected ligand–receptor interactions of MG cells **(E)**, RGC **(F)**, and RPCs **(G)**. Differential transcription pattern of macular and peripheral retinal cells.

The process of vision relies on the cooperation of various cells in the retina, highlighting the importance of understanding the communication between retinal cells. In humans, MGs exhibit the highest frequency of interaction with other cell types (Figure 5A). Conversely, RPCs showed an even higher frequency in mice and zebrafish (Figures 5B,C). Interestingly, we observed that high expression levels of G protein-coupled receptors (*GPR37* and *GPR37L1*) interact with *PSAP*, a highly conserved glycoprotein that induces differentiation and prevents the death of various neuronal cells through an active domain of saposin C (Figures 5D,E) in MGs. This suggests the potential application of MGs in the development of retinal regeneration therapy. Furthermore, MGs may primarily receive signals from RGCs and HCs through neurotrophic tyrosine kinase receptor type 1 (*NTRK1*) (Figure 5E). The *NTRK1* signal, along with the ligand–receptor pair, *FAM3C* and *CXADR*, exhibits striking activity between RGCs and other retinal cells (Figure 5F), indicating that it is involved in the epithelial-to-mesenchymal transition and retinal laminar formation processes (Katahira et al., 2010). Extensive signaling exchange is observed within the Ephs family (Figures 5D,E,G). The *MDK* signal in human MGs was far lower than that in RPCs, whereas the *mdka* signal was active in zebrafish MGs (Supplementary Figure 3B).

### 3.6 Transcriptional differences between macular and peripheral regions

**FIGURE 6:**
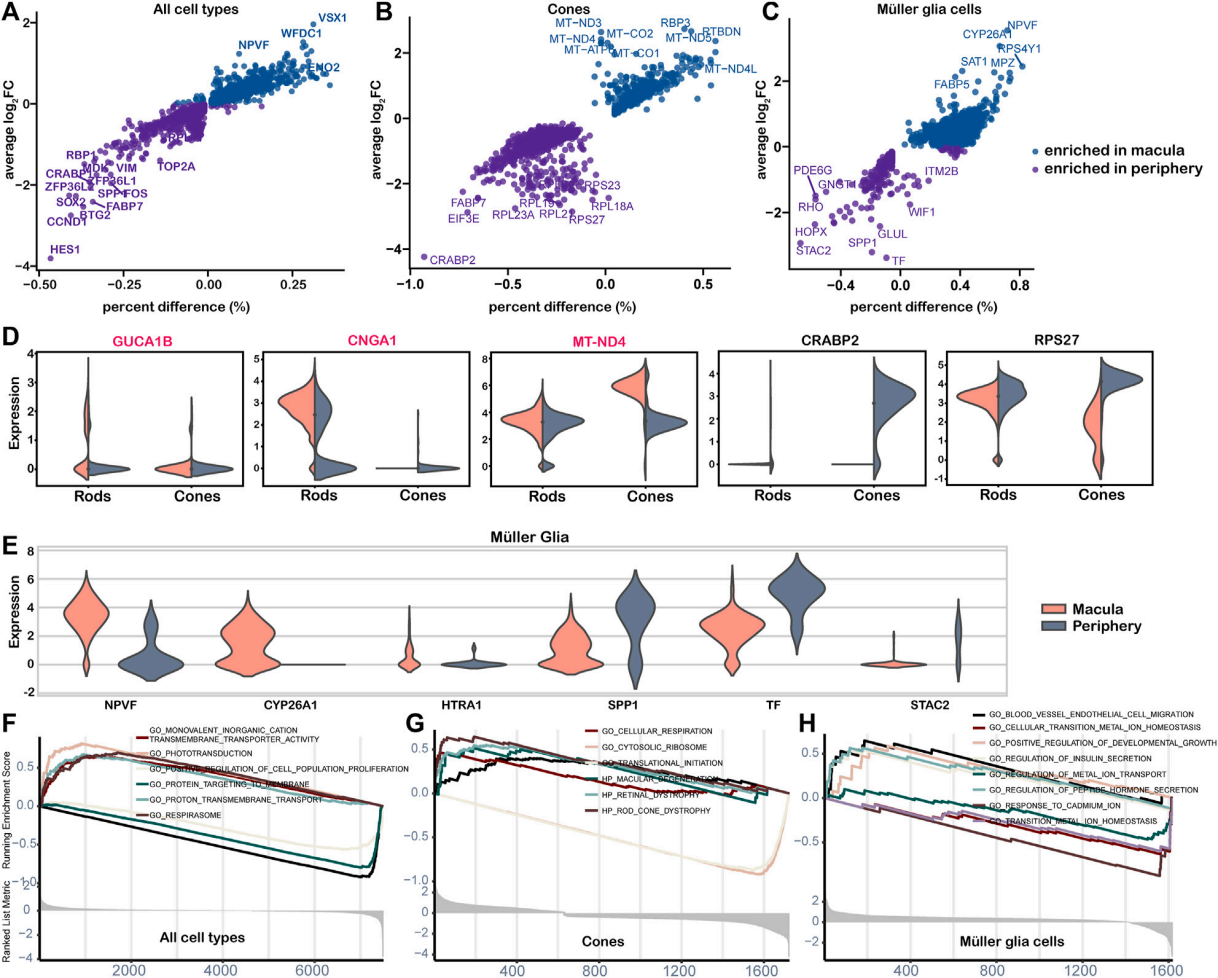
Differential expression analysis of cell clusters by region. **(A–C)** Point plot of cell cluster DE analysis by region. The average log2FC and percent difference of each gene were compared between the two regions. **(D–E)** The violin diagram shows the selected DE genes of the macula and periphery. The red-labeled gene is the IRD gene. **(D)** Gene expressed in rods and cones; **(E)** genes expressed in the Müller glia. **(F–H)** The GSEA plot shows differential pathways in both regions for all cells **(F)**, cones **(G)**, and Müller glial cells **(H)**.

A total of 46 genes showed significantly different expression in the macular and peripheral regions, with 10 genes enriched in the macula and 36 genes enriched in the periphery (Figure 6A, Supplementary Table S5). Among these genes, *HES1*, which confers controlled competence of differentiation (Kageyama et al., 2008), was identified as the most upregulated gene in the periphery. We also observed variation in the abundance of cones and MGs from the macula to the periphery (Figures 6B,C). According to the results, IRD genes *CUCA1B* and *CNGA1* were highly expressed in macular rods, while many mitochondrial genes, including *MT-ND4*, were highly expressed in macular cones, confirming the heightened oxygen consumption and metabolic activity in the macula (Figures 6B,D). *CRABP2*, the highest expression gene in the periphery, functions in transporting retinoic acid to the receptor in the nucleus and regulating cell proliferation and apoptosis (Feng et al., 2019). Furthermore, many ribosomal genes, such as *RPS27*, were highly expressed in peripheral cones, indicating potential active transcription and translation activities (Figure 6B) (Gnanasundram and Fahraeus, 2018). Variations in the gene expression of cones and MGs were also observed from the macula to the periphery (Figures 6B,C,E). Positive regulators of the cell population proliferation term were enriched in the periphery, whereas respirasome and ion transport terms were enriched in the macula (Figure 6F). Consistent with a large number of mitochondrial genes enriched in macular cones, the cellular respiration term was enriched in macular cones from the macula, whereas the translation and cytosolic ribosome term was enriched in the periphery (Figure 6G). The term vascular endothelial cell migration, related to the AMD phenotype, was enriched in macular MGs, while metal ion balance-related terms were enriched in the periphery (Figure 6H).

### 3.7 Evolutionary conservation and divergence along the trajectory of retinal PRs and BCs

**FIGURE 7:**
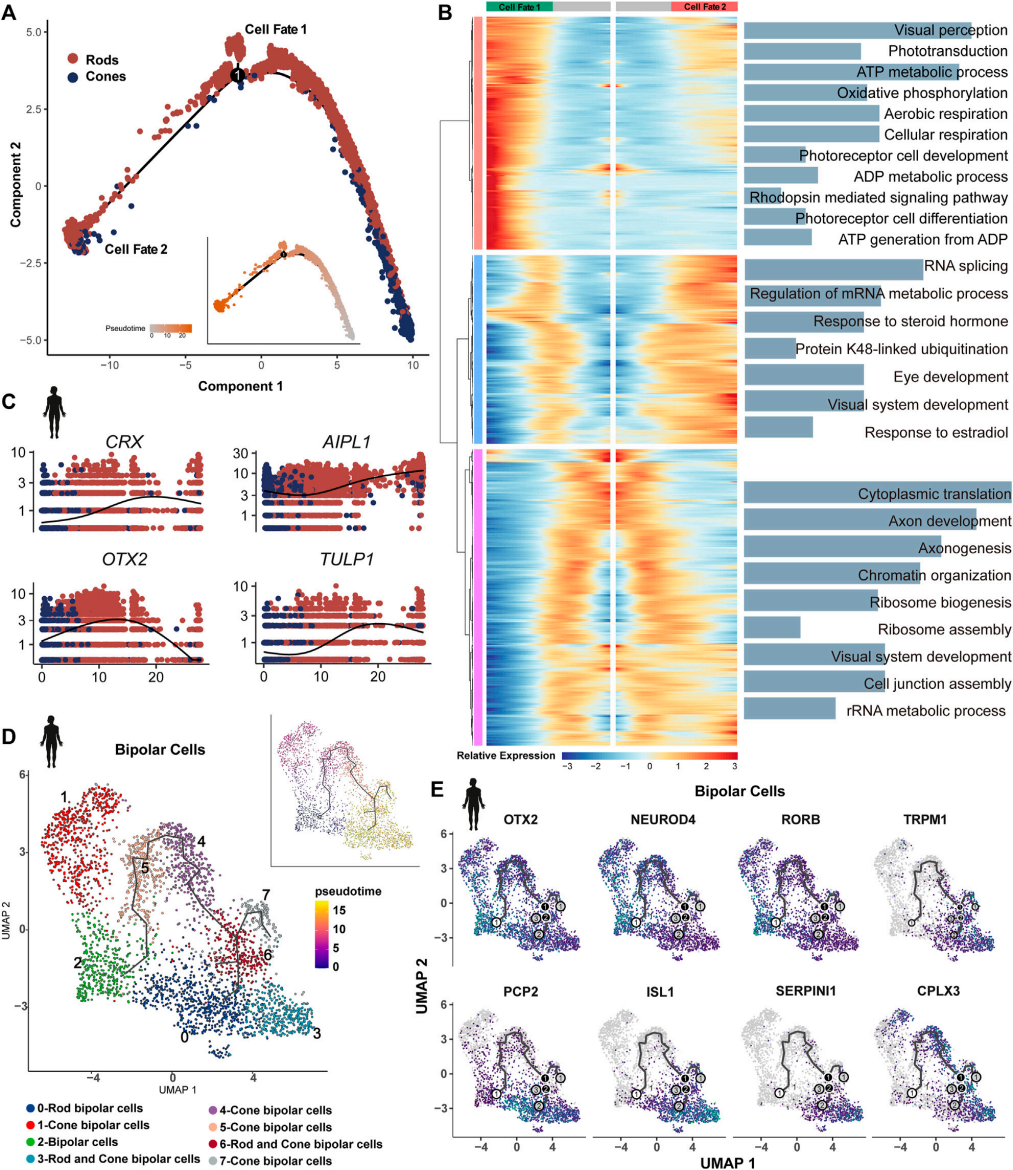
Bifurcation in the transcriptional state of photoreceptors: **(A)** trajectory manifold of photoreceptors from the developing human retina. Cell trajectories/fates were defined by expression profiles. **(B)** Expression heatmap of significant (q < 1e-5) genes based on branch expression analysis comparing the two photoreceptors. GO terms are listed on the right. **(C)** Trace plots showing transcriptional changes in IRD gene expression levels along the pseudotime in the human retina. **(D)** Trajectory manifold of BCs from developing human retinas. **(E)** Feature plot on UMAP of select genes in BCs during development.

The cell trajectory was determined using cones as the initiating cells, with the sample period serving as a reference in humans (Figure 7A). An increase in the expression of genes associated with cellular respiration and oxidative phosphorylation was observed in the PR of cell fate 1 compared to cell fate 2. Both branches showed enrichment in visual system development and the metabolic process (Figure 7B). The same trajectory trend was identified in developing mice (Supplementary Figure 5A). In adult zebrafish and human retinas, PRs are characterized by unbiased trajectory trends (Supplementary Figures 5B,C).

We observed variations in the expression of many IRD genes along the pseudotime in PRs. For example, LCA genes *OTX2* and *AIPL1* exhibited high expression at the beginning, and the LCA gene *LCA5*, Usher genes *ABHD12* and *CIB2*, and CRD genes *RIMS1* and *CNGA3* showed the earliest expression patterns. RP genes *CRX* and *TULP1* exhibited a gradual increase over time, whereas many other RP genes (*RHO*, *RBP3*, *PEX7*, *etc*.) were identified as terminal expression patterns (Figure 7C, Supplementary Figures 5D,E). Our results further support the genetic overlap between LCA and late-onset PR degeneration due to the shared pathogenic genes (Wright et al., 2010). The IRDs (*GNAT2*, *PDEFG*, and *CSPG4*) displayed highly consistent expression in humans and mice (Supplementary Figure 5F). Some IRD genes, including *GNAT1*, *MT-ND4*, and *PDE6A*, exhibited highly dynamic expression during the differentiation of PRs in humans, whereas these patterns were less dynamic in mice.

We re-clustered BCs of human developing retinal samples, generated eight clusters, and annotated them based on the known maker genes. Clusters 4 and 6 consist of a mix of rod and cone BCs. Along the BC trajectory, the clusters containing rod BCs showed a terminal differentiation pattern (Figure 7D). It has been reported that a high level of *OTX2* is associated with the development and differentiation of BCs (Yamamoto et al., 2020). Consistently, *OTX2* (LCA gene) showed high expression at the beginning of the trajectory, as well as in the PR trajectory. We also observed similar expression patterns for *NEUROD4* and *RORB*, suggesting their potential role in the development and differentiation of BCs (Figure 7D). In contrast, CSNB gene *TRPM1* showed terminal expression patterns, which are essential for the development of rod BCs and their synaptic connections with subsequent neurons (Kozuka et al., 2017). Similarly, we identified that *PCP2*, *ISL1*, *SEPINI1*, and *CPLX3* exhibited terminal mode characteristics (Figure 7E). *PCP2* was highly expressed in rod BCs at the end of trajectory. The retina-specific splice variant of *PCP2*, Ret-PCP2, accelerates the light response of rod BCs by modulating the mGluR6 transduction cascade (Sulaiman et al., 2010). In mice, *Isl1* orchestrates the early differentiation and maintenance of various cell types in the retina across different vertebrates (Bejarano-Escobar et al., 2015).

## 4 Discussion

Analysis at the single-cell resolution expanded our understanding of cellular functions in various tissues, including the retina. In this study, we presented a comprehensive overview of cell types and subpopulations in 35 retinal samples from three species at single-cell resolution. By assessing the molecular changes and different signaling profiles of IRD genes of different cell types, investigating the role of IRDs in the regulatory network of the retina, and inferring IRD gene traits along PR and BC trajectories, our results provided a strong basis for the clinical diagnosis and pathological mechanism exploration of subsequent IRDs.

Here, we found that retinal homogeneous cells performed shared-transcriptome patterns in different species, with species-specific idiosyncrasies (Figure 1). As the recent study reported, the similarity in expression patterns between RGCs and HCs could be attributed to their close developmental relationship (Yi et al., 2021). The highly expressed regenerative genes including *atoh7* and *mdkb* were observed in zebrafish rather than in mice and humans, which indicated the unique regenerative ability of the zebrafish retina and provided a reference for developing a targeted therapy for IRDs. Moreover, we also found species-specific signals among the retinal cells of different species. In zebrafish, *mdka* (*MDK* homolog) is upregulated in the stem cell niche and by MGs during reprogramming to neurogenic progenitor cells (Gramage et al., 2014). Thus, the high conservation of IRD genes suggest the importance of these genes in retinal structure and function, while the selection of model organisms should take into account the differential expression of IRD genes.

In this study, we observed that IRD genes were more abundantly enriched in MGs than in other cells, which is consistent with previous reports (Yan et al., 2020). Furthermore, we also found that MGs exhibited the highest frequency of cellular interaction among species. These findings suggested that IRD genes were enriched in terms related to cell homeostasis and metabolism (Supplementary Figure 1C and Supplementary Table S2). The role of MGs in the regulation of the extracellular space volume and ion and water homeostasis is crucial for IRDs (Reichenbach and Bringmann, 2020).

AMD is a progressive degenerative disease affecting the macula (Deng et al., 2022). Our observations reveal that genes in the complement pathway, such as *C2*, *C3*, and *CFB* involved in AMD, showed high expression in MGs. The last decade has seen the development of several novel therapeutics targeting the complement pathway in the eye for the treatment of AMD (Tzoumas et al., 2023). *VEGFA* was identified as the target gene of the MG-specific regulon. Complement inhibitors and anti-*VEGF* were tried as the treatments for AMD (Amoaku et al., 2015; Tzoumas et al., 2023). Additionally, an enrichment of the term blood vessel endovascular cell migration was observed in the macula MGs (Figures 6B,H), which is consistent with PR degeneration triggered by geographic atrophy or choroidal neovascularization. The mitochondrial gene enrichment in the macular cones further supports the role of oxidative stress in the pathogenesis of AMD at the expression level. These findings suggest that the molecular functional exploration of MGs in the macula could serve as a basis for the clinical diagnosis of AMD.

Our results support the genetic overlap between LCA and late-onset PR degeneration, as well as a clinical and genetic overlap between RPs and CRDs, which increases the complexity of diagnosis and treatment (Wright et al., 2010; Varin et al., 2021). Rodent (*Crb1*, *Lrat*, *Mertk*, *Rpe65*, and *Rpgrip1*), avian (*Gucy2D*), and canine (*Rpe65*) models for LCA and profound visual impairment have been successfully corrected employing adeno-associated virus or lentivirus-based gene therapy (den Hollander et al., 2008). Our findings can provide a theoretical basis for enhancing LCA gene therapies at the single-cell level. One notable aspect is the degeneration sequence of rods and cones in the retinas. In our results, most RP genes exhibited a higher expression level in rods and MGs. For instance, RP genes were identified in rod-specific *CRX*/*RAX2* regulons. In contrast, CRD genes showed a high expression pattern specifically in cones. The expression trajectories of these genes may explain this phenomenon. Through trajectory inference of PRs, RP genes exhibited progressive characteristics or terminal expression patterns, whereas CRD genes only displayed early-onset traits (Figure 7C, Supplementary Figures 5D). Particularly, the LCA gene *OTX2* exhibited a high expression level at the beginning in both PRs and BCs. These results aim to analyze the subtle variances between RPs and CRDs, LCA, and late-onset PRs at a single-cell resolution, offering a solid scientific foundation for clinical diagnosis.

We also discovered that the CSNB genes predominantly existed in rod and BC-specific regulons and showed significant expression levels in both rods and BCs, consistent with the phenotype of disrupted signaling between PRs and BCs. It is hypothesized that the genes in the *OTX2* regulon attributed to BCs and rods could potentially serve as candidate genes for CSNB. The specific intercellular communication from PRs to BCs cannot be ignored, which might be the crucial ligand–receptor pairs that mediate signaling pathways relevant to the pathogenesis of CSNB.

Optic atrophy (OA), the most prevalent inherited optic neuropathy encountered in clinical practice, also showed specificity to the macula. The characteristic pathology involves the production of excessive ROS due to mitochondrial dysfunction, serving as a trigger for RGC apoptosis (Chun and Rizzo, 2017). There are increasing efforts to develop strategies for the effective reduction of RGC death, including the inhibition of pro-apoptotic signaling, modulation of the inflammatory response, and neurotrophic factor delivery (Kole et al., 2020). It would not be surprising to discover new IRD genes or potential therapeutic targets in ligand–receptor pairs associated with RGCs.

Our results indicate that the phenotype of IRDs is caused by dysfunction in certain cells, characterized by the unique expression of specific sub-gene sets, including cellular expression, regional expression, and TF regulons. Some IRD genes were identified in the subtypes of IRDs as a unit of regulons. The application of clustering techniques offers a valuable and systematic method to study IRDs.

### 4.1 Limitations of the study

The tissue characteristics of the retina make it extremely difficult to obtain both healthy adult samples and diseased adult samples from humans, and the lack of patient samples also makes it impossible for us to verify the observation results with patient samples at the single-cell levels. With the advancement of iPSC and retinal organoid technology, it is expected that the difficulty of obtaining adult retinal samples can be greatly improved in the future. In addition, while most cell types are balanced among samples, it is difficult to distinguish physiologically significant processes or technical effects. The intrinsic sparsity of scRNA-seq data makes it difficult to annotate cell types with low RNA levels. In the future, enrichment of single-cell references can help refine labeling for less common cell types.

## 5 Conclusion

To the best of our knowledge, this study represents the initial attempt to construct the co-expression network, regulatory network, and cell–cell communication network to infer the role of IRD genes. In our findings, most IRD genes, especially the RP genes, were enriched in rod-specific regulons. In addition, we found that CSNB genes *GRK1*, *PDE6B*, and *TRPM1* showed cell-specific expression and transcription characteristics in either rods or BCs, which were consistent with the differentially expressed IRD genes of different cell types. The spatiotemporal expression patterns of genes, along with their module and regulon results, can provide valuable insights into the pathogenesis of IRDs and provide potential target information for the treatment and intervention of these degenerative retinal diseases. IRD genes exhibited both evolutionary conservation (*GNAT2*, *PDE6G*, and *SAG*) and divergence (*GNAT2*, *MT-ND4*, and *PDE6A*) along the PR trajectory across species. For example, the LCA gene *OTX2* was highly expressed at the start of both PRs and BCs. This is essential for grasping the underlying disease mechanisms and enhancing our understanding of the connections between genes and phenotypes, as well as the cellular and molecular processes that drive this heterogeneity. It forms a solid basis for potential treatment strategies.

## Data Availability

Existing datasets are available in a publicly accessible repository: Publicly available datasets were analyzed in this study. This data can be found here: GEO database (www.ncbi.nlm.nih.gov/geo/): GSE138002, GSE118614, GSE132229, GSE122680 and GSE160140; ArrayExpress (https://www.ebi.ac.uk/arrayexpress/): E-MTAB-7316.

## Abbreviations

IRDs, inherited retinal diseases; LCA, Leber congenital amaurosis; CSNB, congenital stationary night blindness; RP, retinitis pigmentosa; CRDs, cone–rod dystrophies; MD, macular degeneration; RPCs, retinal progenitor cells; HCs, horizontal cells; cones, cone photoreceptor cells; ACs, amacrine cells; rods, rod photoreceptor cells; BCs, bipolar cells; MGs, Müller glias; TFs, transcription factors; GEO, Gene Expression Omnibus; UMI, unique molecular identifier; PCA, principal component analysis; tSNE, t-distributed stochastic neighbor embedding; GO, Gene Ontology; GSEA, Gene Set Enrichment Analysis; WGCNA, weighted gene co-expression network analysis; GS, gene significance; MM, module membership; SCENIC, Single-Cell rEgulatory Network Inference and Clustering; DEGs, differentially expressed genes; PRs, photoreceptors.
