# Supplementary figures and images for: Cross-species single-cell landscapes identify the pathogenic gene characteristics of inherited retinal diseases
Source: Front Genet. 2024 Jul 11;15:1409016. doi: 10.3389/fgene.2024.1409016 (PMC11269129; doi:10.3389/fgene.2024.1409016)

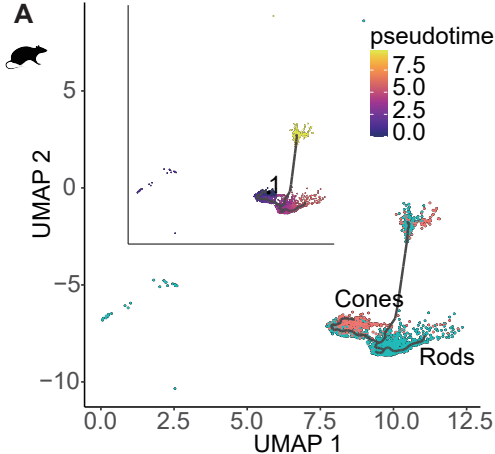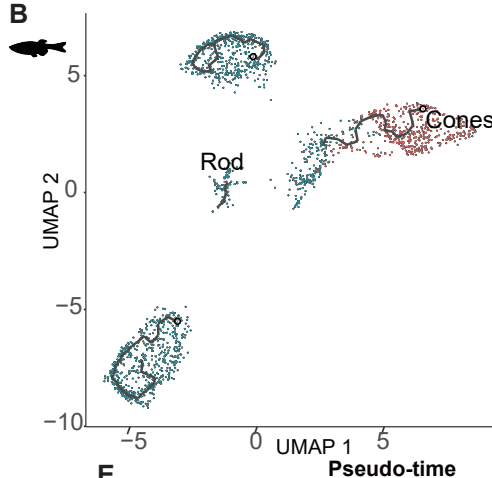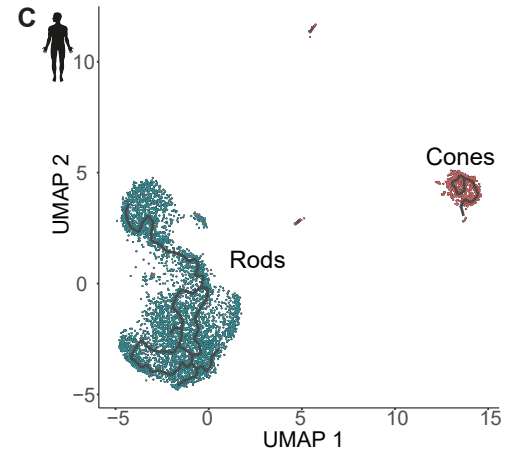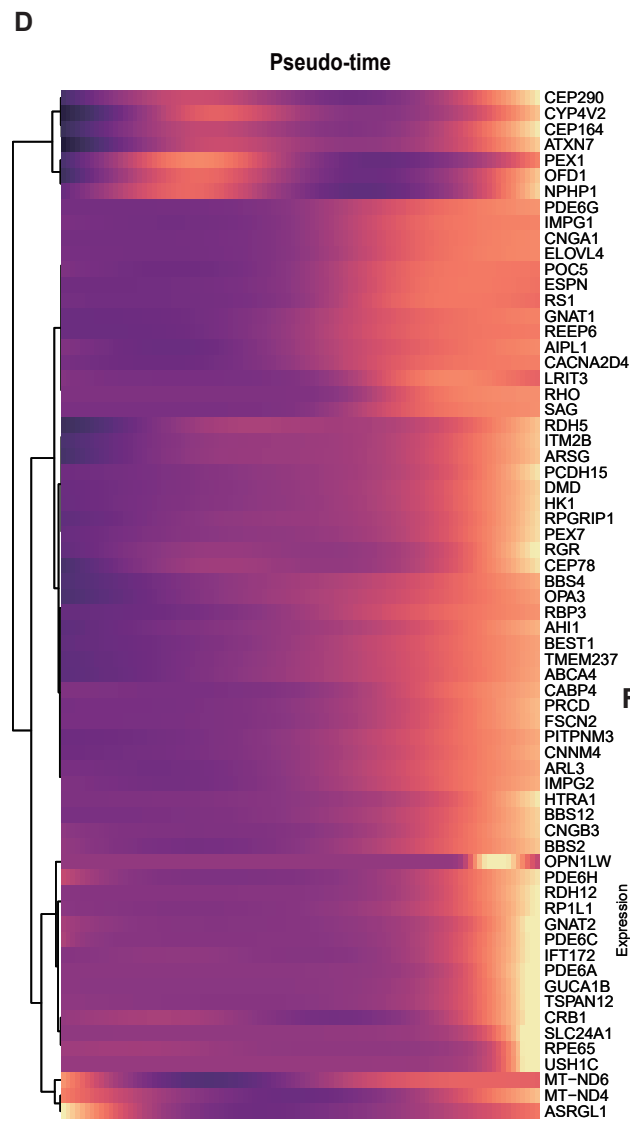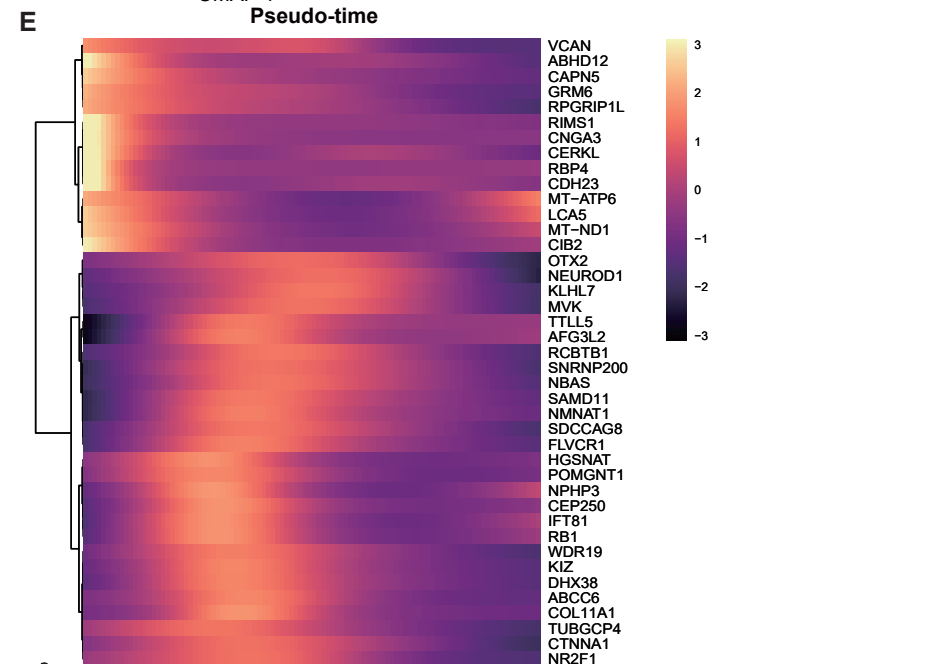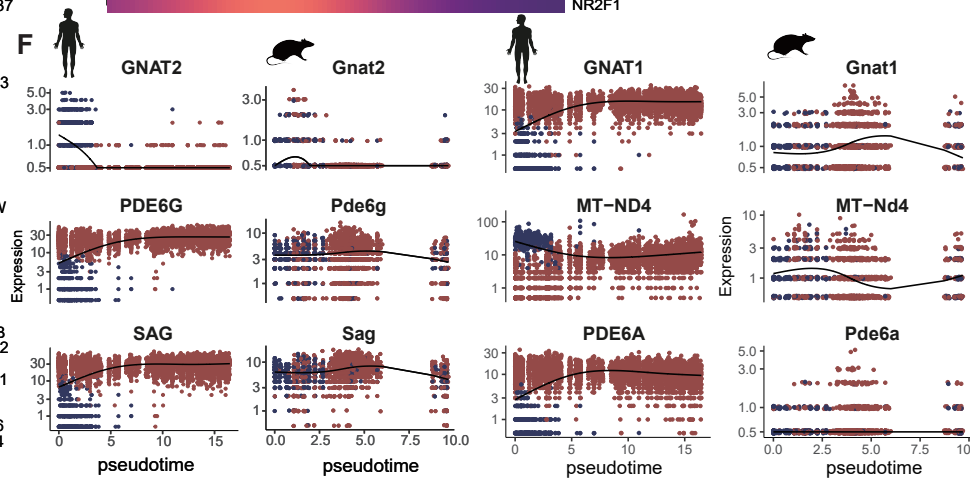

Supplement: Supplementary file 3 [file Image5.PDF]

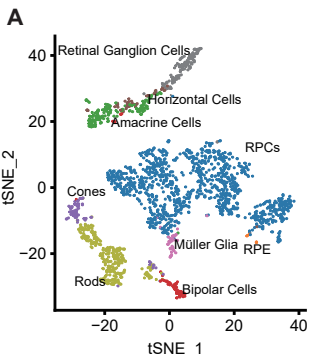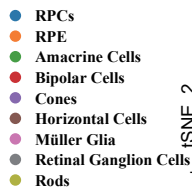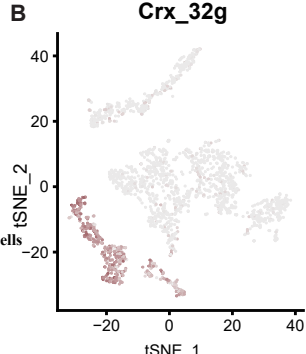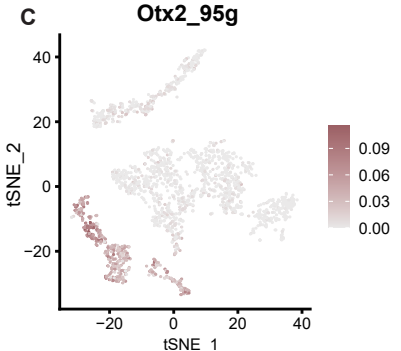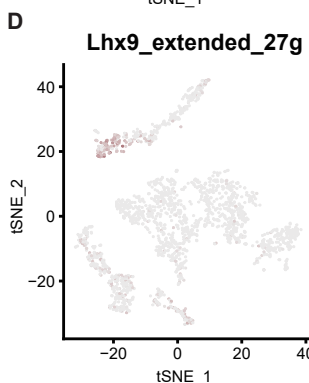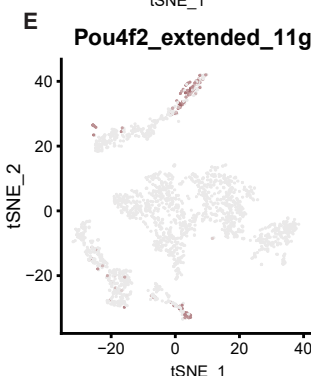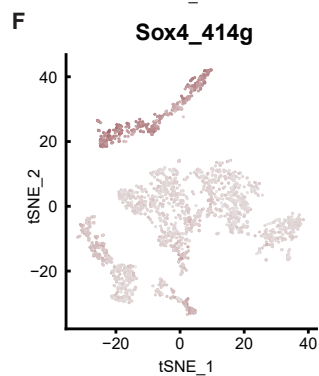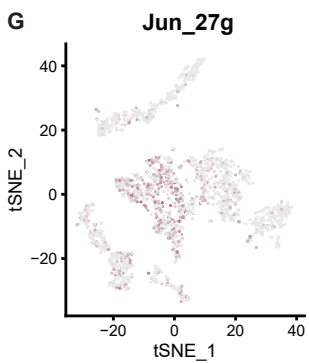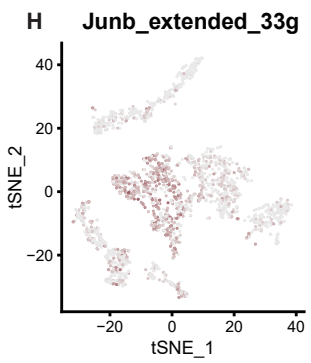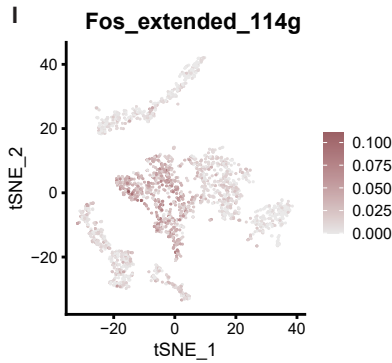

Supplement: Supplementary file 4 [file Image4.PDF]

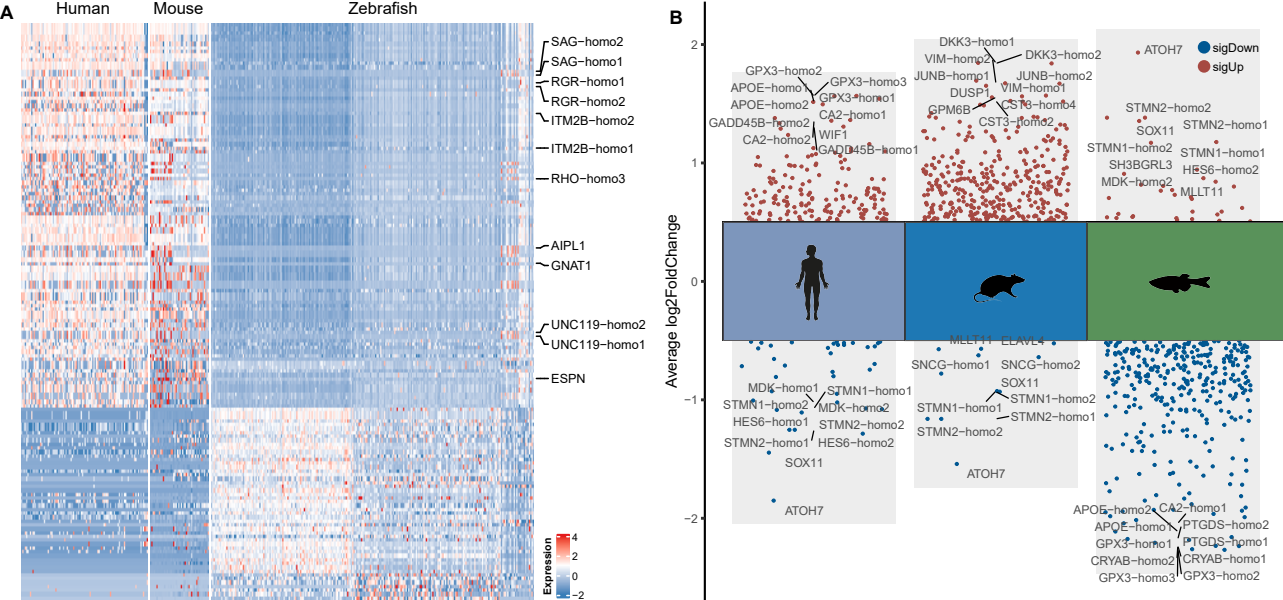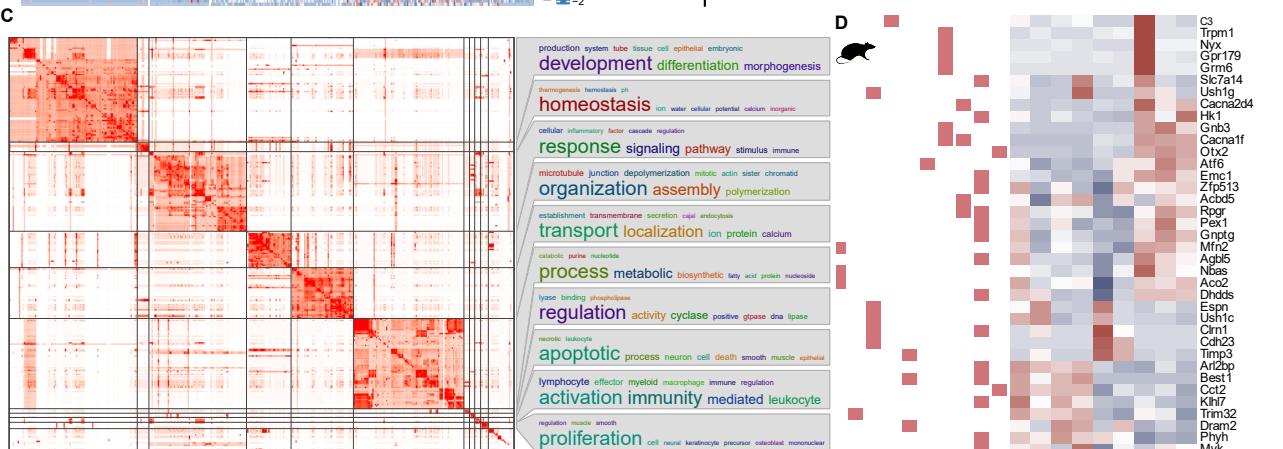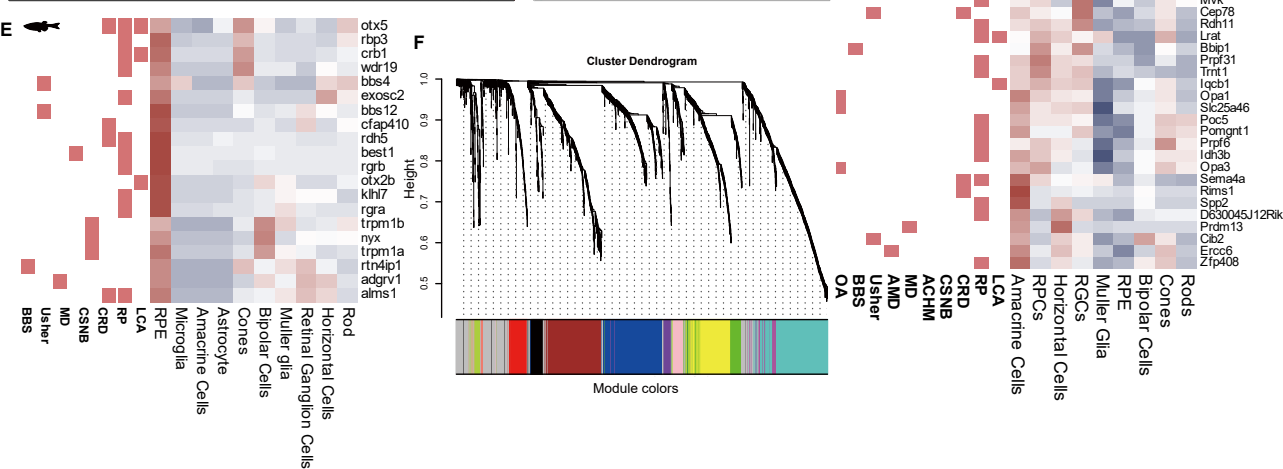

Supplement: Supplementary file 5 [file Image2.PDF]

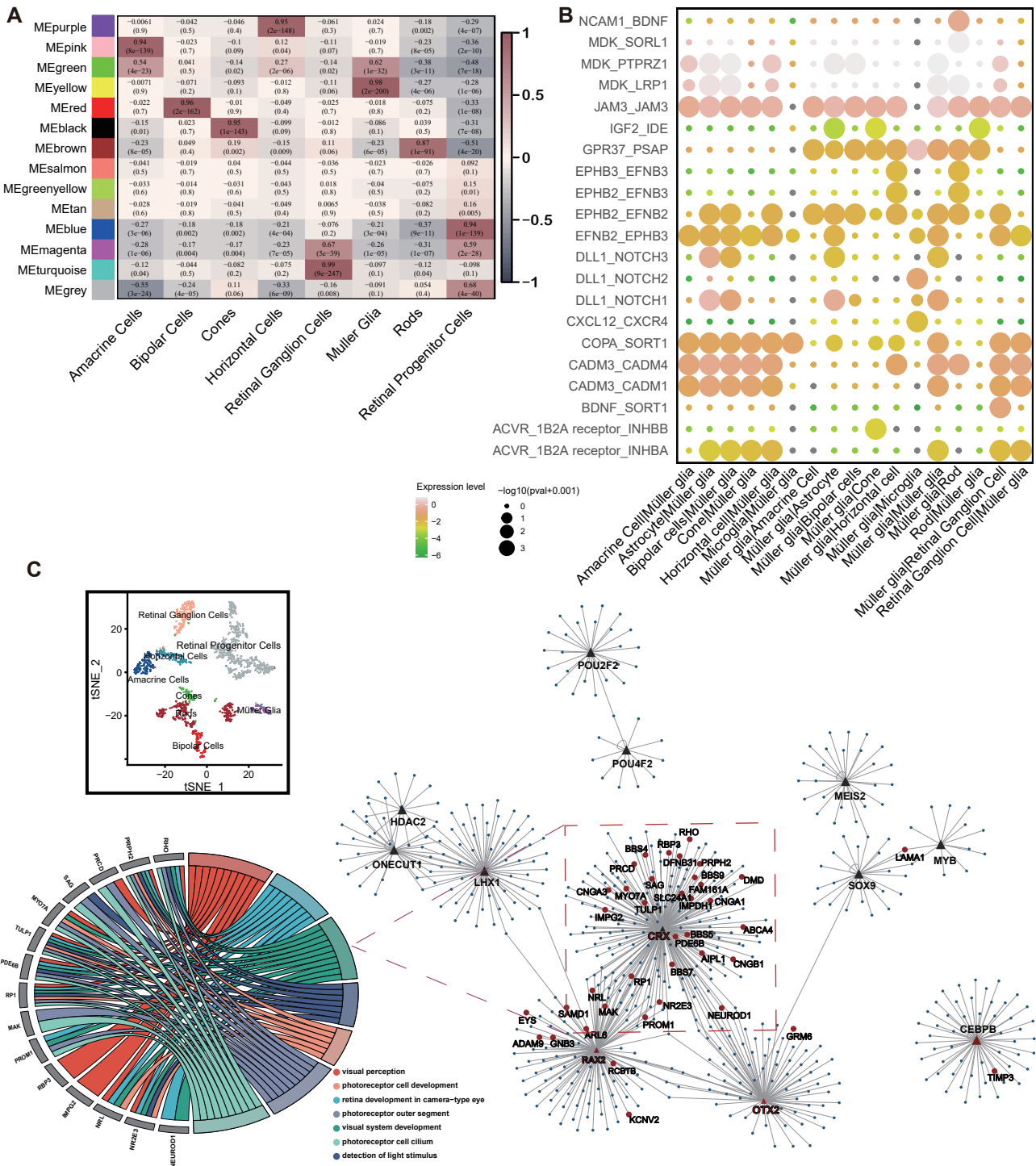

Supplement: Supplementary file 6 [file Image3.PDF]

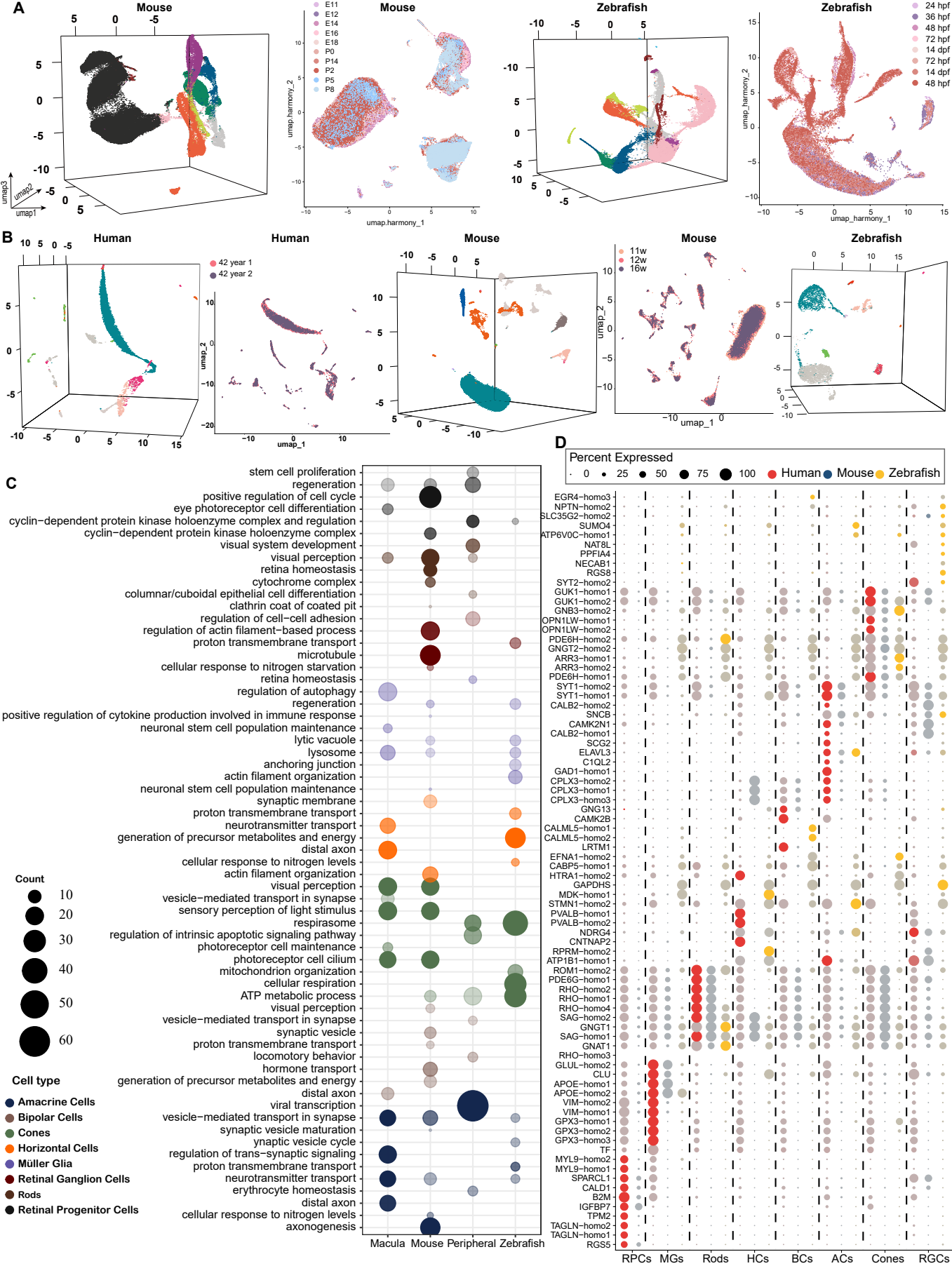

Supplement: Supplementary file 11 [file Image1.PDF]
