# Supplementary material for: Cross-species single-cell landscapes identify the pathogenic gene characteristics of inherited retinal diseases
Source: Front Genet. 2024 Jul 11;15:1409016. doi: 10.3389/fgene.2024.1409016 (PMC11269129; doi:10.3389/fgene.2024.1409016)
Supplement: Supplementary file 9 [file Table6.DOCX]

**Key resources table**

| **Regent or Resource** | **Source** | **Identifier** | **Reference** |
| --- | --- | --- | --- |
| **Deposited data** |  |  |  |
| developing human retina | GEO | GSE138002 | (Lu et al., 2020) |
| adult human retina | ArrayExpress | E-MTAB-7316 | (Lukowski et al., 2019) |
| developing mouse retina | GEO | GSE118614 | (Clark et al., 2019) |
| adult mouse retina | GEO | GSE132229 | (Heng et al.) |
| developing zebrafish retina | GEO | GSE122680 | (Xu et al., 2020) |
| adult zebrafish retina | GEO | GSE160140 | (Liu et al., 2022) |
| IRD genes | RetNet (https://sph.uth.edu/retnet/) |  |  |
| Homologous gene | BioMart (www.ensembl.org/) |  |  |
| **Software and algorithms** |  |  |  |
| R | https://www.r-project.org/ | v4.3.2 |  |
| RStudio | https://www.rstudio.com/ | v2023.06.2-561 |  |
| Python | https://www.python.org/ | v3.8.18 |  |
| Anaconda3 | https://www.anaconda.com | v23.7.3 |  |
| Seurat | https://github.com/satijalab/seurat | v5.0.1 | (Hao et al., 2024) |
| Harmony |  |  | (Korsunsky et al., 2019) |
| clusterProfiler | https://github.com/YuLab-SMU/clusterProfiler | v4.6.2 | (Yu et al., 2012) |
| scWGCNA | https://github.com/CFeregrino/scWGCNA | v0.3.01 | (Langfelder and Horvath, 2008) |
| Cytoscape | https://cytoscape.org | v3.9.0 | (Shannon et al., 2003) |
| pySCENIC | https://github.com/aertslab/pySCENIC | v0.12.1 | (Aibar et al., 2017) |
| CellPhoneDB | https://github.com/Teichlab/cellphonedb | v5.0.0 | (Efremova et al., 2020) |
| monocle | https://github.com/cole-trapnell-lab/monocle-release | v2.24.0 | (Trapnell et al., 2014) |
| monocle3 | https://github.com/cole-trapnell-lab/monocle3 | v1.3.1 | (Qiu et al., 2017) |
